# The effect of omega-3 long chain polyunsaturated fatty acids on aggressive behaviour in adult male prisoners: a structured study protocol for a multi-centre, double-blind, randomised placebo-controlled trial and translation into policy and practice

**DOI:** 10.1186/s13063-021-05252-2

**Published:** 2021-05-02

**Authors:** Barbara J. Meyer, Mitchell K. Byrne, Colin H. Cortie, Natalie Parletta, Alison Jones, Simon Eckermann, Tony Butler, David Greenberg, Marijka Batterham, Francesca Fernandez, Peter W. Schofield, Pia C. Winberg, Kate Bowles, Jean Dally, Anne-Maria Martin, Luke Grant

**Affiliations:** 1 School of Medicine, Lipid Research Centre, Molecular Horizons, University of Wollongong and Illawarra Health & Medical Research Institute (IHMRI), Northfields Ave, Wollongong, NSW 2522 Australia; 2 School of Psychology, University of Wollongong, Northfields Ave, Wollongong, NSW 2522 Australia; 3 Centre for Population Health Research, University of South Australia, GPO Box 2471, Adelaide, South Australia 5001 Australia; 4 DVC (Health and Communities), University of Wollongong, Northfields Ave, Wollongong, NSW 2522 Australia; 5 Australian Health Services Research Institute, University of Wollongong, Northfields Ave, Wollongong, NSW 2522 Australia; 6 Kirby Institute, University of New South Wales, Sydney, NSW 2052 Australia; 7 School of Psychiatry, University of New South Wales, Sydney, NSW 2052 Australia; 8 Justice Health & Forensic Mental Health Network, Anzac Pde., Matraville, NSW 2035 Australia; 9 School of Mathematics & Applied Statistics, University of Wollongong, Northfields Ave, Wollongong, NSW 2522 Australia; 10 School of Health and Behavioural Science, Australian Catholic University, Banyo, QLD 4014 Australia; 11 School of Medicine and Public Health, University of Newcastle, Newcastle, NSW 2300 Australia; 12 Venus Shell Systems, PO Box 2149, Bomaderry, NSW 2541 Australia; 13 School of the Arts, English and Media, University of Wollongong, Northfields Ave, Wollongong, NSW 2522 Australia; 14 Corrective Services NSW, 20 Lee St, Haymarket, NSW 2000 Australia

**Keywords:** Omega-3, Aggressive behaviour, Attention deficit disorder, Mental health, Violence, Correctional centres, Jails, Incarcerated, Prisoner, Inmate

## Abstract

**Background:**

Interventions to better manage aggressive behaviour and reduce recidivism are a primary concern for corrective services. Nutritional interventions to correct prisoner behaviour have been largely overlooked in the literature. Emerging evidence suggests that dietary intake influences aggressive behaviours and that nutritional supplementation with omega-3 long chain polyunsaturated fatty acids (n-3 LCPUFA) could attenuate both the severity and frequency of aggressive behaviour.

**Methods:**

Adult male prisoners who have a history of aggressive behaviour (*n* = 600) will be recruited from at least 6 Correctional Centres and randomised to receive either n-3 LCPUFA or placebo supplementation for a 16-week duration. Treatment will be with either 1 g/day of n-3 LCPUFA (694 mg DHA and 397 mg EPA) or placebo capsules, which are a corn/soy oil blend and are identical in size and colour.

The primary outcome measure is the Inmate Behavioural Observation Scale (IBOS): an objective measure of aggressive behaviour. Secondary outcome measures include questionnaires (including aggression, attention deficit disorder, impulsivity, depression/anxiety/stress scales), engagement in programmes, recidivism and quality of life. Baseline and post-intervention assessments include the IBOS, questionnaires and blood to measure the levels of n-3 LCPUFA.

**Discussion:**

To conclusively test the potential that increasing n-3 LCPUFA intakes can improve rates of prisoner aggression and associated mental health and violence-related social system management costs, we propose an adequately powered multi-centre, double-blind, randomised controlled trial, examining the effects of n-3 LCPUFA supplementation on aggressive behaviour in adult male prisoners. If successful, this study will inform prisoner policy with respect to nutrition and by inference contribute to a broader community approach to preventative mental health practices.

**Trial registration:**

Australian New Zealand Clinical Trial Registry (ANZCTR) ACTRN12618001665224. Registered on 10 October 2018.

## Administrative information

**Table:** 

Title {1}	The effect of omega-3 long chain polyunsaturated fatty acids on aggressive behaviour in adult male prisoners: a structured study protocol for a multi-centre, double blind, randomised placebo controlled trial and translation into policy and practice
Trial registration {2a and 2b}.	The trial is registered in the Australian New Zealand Clinical Trial Register (http://www.anzctr.org.au), ACTRN12618001665224
Protocol version {3}	The protocol version number is OmegaMan2018-002, version 6, 24 August 2018.
Funding {4}	NHMRC Partnership Grant (GNT1113396) Does Omega-3 Supplementation Attenuate Aggressive Behaviour: A Multi-Centre Randomised Controlled Trial of a Broadly Disseminable Strategy. The partner organisations are: DSM Nutritional Products and Correctional Centres in NSW and the Department for Correctional Services SA. DSM Nutritional Products provided the supplements (active and placebo) as well as cash for this research
Author details {5a}	Barbara J. Meyer^1*^, Mitchell K. Byrne^2^, Colin H. Cortie^1^, Natalie Parletta^3^, Alison Jones^4^, Simon Eckermann^5^, Tony Butler^6^, David Greenberg^7,8^, Marijka Batterham^9^, Francesca Fernandez^10^, Peter Schofield^11^, Pia C. Winberg^12^, Kate Bowles^13^, Jean Dally^14^, Anne-Maria Martin^14^, Luke Grant^14^ ^1^School of Medicine, Lipid Research Centre, Molecular Horizons, University of Wollongong, Illawarra Health & Medical Research Institute (IHMRI), Northfields Ave, Wollongong, NSW 2522 Australia. ^2^School of Psychology, University of Wollongong, Northfields Ave, Wollongong, NSW 2522 Australia. ^3^Centre for Population Health Research, University of South Australia, GPO Box 2471, Adelaide, South Australia, 5001, Australia. ^4^DVC (Health and Communities), University of Wollongong, Northfields Ave, Wollongong, NSW 2522 Australia. ^5^Australian Health Services Research Institute, University of Wollongong, Northfields Ave, Wollongong, NSW 2522 Australia ^6^Kirby Institute, University of New South Wales, Sydney, NSW 2052, Australia. ^7^School of Psychiatry, University of New South Wales, Sydney, NSW 2052, Australia. ^8^Justice Health & Forensic Mental Health Network, Anzac Pde., Matraville, NSW 2035, Australia ^9^School of Mathematics & Applied Statistics, University of Wollongong, Northfields Ave, Wollongong, NSW 2522 Australia ^10^School of Health and Behavioural Science, Australian Catholic University, Banyo 4014, Queensland, Australia ^11^School of Medicine and Public Health, University of Newcastle, Newcastle, NSW, 2300, Australia ^12^Venus Shell Systems, PO Box 2149, Bomaderry, NSW 2541, Australia ^13^School of the Arts, English and Media, University of Wollongong, Northfields Ave, Wollongong, NSW 2522 Australia. ^14^Corrective Services NSW, 20 Lee St, Haymarket NSW 2000, Australia
Name and contact information for the trial sponsor {5b}	Ms Sharon Hughes, Research Services Office, The University of Wollongong, Northfields Avenue, Wollongong, New South Wales (NSW) 2522 Australia.
Role of sponsor {5c}	The role of the funding body, the National Health and Medical Research Council is responsible for providing cash and checking that research milestones are met. The role of DSM Nutritional Products is to supply the omega-3 (algal oil) and placebo oil capsules as in kind support, plus provide a substantial cash contribution for the research but they have no input into the design of the study and collection, analysis and interpretation of data and reporting of results. The role of Correctional Centres in NSW and SA are to provide in kind support of human resources, project management support, office & materials, travel and accommodation costs.

## Introduction

### Background and rationale {6a}

While the importance of good nutrition for optimal mental health is increasingly being recognised [1], poor nutrition and low educational attainment are more common in low socio-economic groups and both are defining characteristics of prisoner populations. A vast body of literature has attested to the benefits of dietary supplementation on mental health issues in general [1] and an emerging literature suggests that dietary supplementation may also impact upon aggressive behaviour [2, 3]. Nutrition is rarely supplemented as a strategy for improving prison behavioural outcomes, even as part of existing mental health intervention programmes which tend to focus on acute needs rather than long-term management [4]. Given substantial societal costs associated with violence and the potential for violence prevention with a simple, accessible and low-cost dietary modification among those at elevated risk of these behaviours, rigorous studies to establish an evidence base are required. The proposed study is designed to contribute to that broad endeavour.

#### Why n-3 LCPUFA?

Omega-3 long chain polyunsaturated fatty acids (n-3 LCPUFA) have cardiovascular health benefits [5, 6], and there is emerging evidence for n-3 LCPUFA benefits for mental health benefit, including reducing violent or aggressive behaviour [7].

n-3 LCPUFA were discovered in the 1970s when researchers set out to investigate why Inuit people of Greenland had high consumption of fat via whale, seal and fish yet low incidence of cardiovascular disease (CVD) [8]. Their findings were extended to other populations that had high fish consumption and low CVD incidence [9, 10], and n-3 LCPUFA have since been associated with numerous health benefits. However, it is well established that n-3 LCPUFA are specifically and substantially deficient in Australian (‘Western’) diets [11, 12]. This is due to relatively low consumption of fish/seafood, the richest sources of n-3 LCPUFA, in particular the semi-essential eicosapentaenoic acid (EPA) and docosahexaenoic acid (DHA). The development of an ‘omega-3 index’ (erythrocyte EPA + DHA levels as percent of total fatty acids) [13], pooling results from numerous trials in cardiac disease, showed that an index ≤4% was associated with the greatest risk for cardiac mortality while ≥8% conferred the greatest protection. In Australia, the average index is 5% [14, 15].

#### Addressing the n-3 LCPUFA controversy

Randomised controlled trials and meta-analysis of trials up to 2002 demonstrated that supplementation with n-3 LCPUFA resulted in up to 45% reduction in overall mortality from cardiac death [5, 16]. However, reviews and meta-analyses of more recent trials reported a lack of efficacy, questioning whether increased n-3 LCPUFA levels improve health outcomes [17, 18]. Nevertheless, critical analyses of these recent trials have suggested that their observed lack of efficacy may have been due to methodological problems arising across populations studied [19]. Following the earlier successful trials, the American Heart Association issued guidelines for people with heart disease, suggesting consumption of at least 2 fish meals per week [20] in addition to fish oil supplements [21]. Between 2000 and 2010, the importation of fish oils into the USA escalated more than 10-fold from 2000 to 22,000 metric tonnes [22]. However, trials conducted after 2002 failed to screen people for high fish and/or fish oil supplement intake resulting in great variability of n-3 LCPUFA status across trial conditions [19]. Where screening did occur, the upper 50% of the control subjects and the lower 50% of the fish oil intervention subjects overlapped [23], suggesting that the 2 groups were not separated enough in use of fish oils to demonstrate an effect in the test arm (n-3 LCPUFA). Thus, robust trials of using fish oil to address omega-3 deficiency need to attenuate possible ceiling effects created by high baseline levels, by taking blood samples to determine compliance and correlations between increased omega-3 index and response. This is supported by the International Society for the Study of Fatty Acids and Lipids (ISSFAL) recent position statement, which highlights the importance of measuring blood n-3 LCPUFA levels in research [24].

#### n-3 LCPUFA and mental health

Both EPA and DHA are important for optimal brain function and have different mechanisms of action [25]. DHA is a structural component of neurons, affecting both physicochemical properties of the neuronal membrane (such as membrane fluidity and permeability) but also neurotransmission through gene expression modulation, neuronal enzyme activity and postsynaptic receptors [26, 27]. Due to its involvement in the regulation of the central neurotransmission such as dopamine and serotonin, DHA along with EPA administration seems beneficial in reducing depressive symptoms [28]. EPA is not concentrated in neural cell membranes but it may have neuro-immunological and vascular effects [25, 29]. EPA competes with the omega-6 arachidonic acid for the production of eicosanoids, and the EPA-derived eicosanoids have anti-inflammatory, anti-thrombogenic and vasodilatory effects resulting in increased blood flow in the brain and improved neuroprotection [25, 29]. It is well established that DHA plays an important role in neurological development and suboptimal levels are associated with mental health issues such as hyperactivity, poor impulse control and depression [7] and low omega-3 index (an average of 4.1% [30] and an average of 3.0% in people at ultra-high risk of psychosis [31]) is associated with increased mental illness [30, 32]. Therefore, a body of research has investigated the role of n-3 LCPUFA acids in mental health [2, 25, 33].

#### n-3 LCPUFA, aggression and violent behaviour

n-3 LCPUFA may be important in controlling aggression/hostility and self-harm [32, 34, 35]. Significant decreases in anger and anxiety in a population of substance abusers were reported after supplementation of EPA and DHA for 3 months [36]. EPA has also been shown to reduce antisocial and aggressive behaviours [37]. Eleven out of 14 randomised controlled trial studies reported a positive effect of fish oils on aggression/hostility [35].

#### Inmate Behaviour Observation Scale (IBOS)

The IBOS was developed by (Byrne MK, Cortie CH, Meyer BJ. The Inmate Behaviour Observation Scale (IBOS): A measure of prisoner aggression and violence based on inmate case notes, in preparation/forthcoming) and is an observation-based measure of aggressive behaviour based on inmate case notes. The IBOS was developed to overcome the different environments across different Correctional Centres, and it has been shown to have construct validity and inter-rater reliability. The IBOS is an ideal tool to use in multi-centre trials.

#### Prisoner populations, mental health and violence

The logistics of conducting intervention trials in corrective services settings are challenging, and hence, only two supplementation studies [2, 3] have been published to date in Europe in adult male prisoners. Both of these well-controlled studies showed that, with nutritional supplementation of multivitamins/minerals and n-3 LCPUFA, there was a 26–35% reduction in the number and severity of in-prison reprimands, particularly for violent behaviour [2, 3]. However, both of these studies had two significant limitations. First, neither of these studies acquired blood samples to measure the changes in n-3 LCPUFA and hence these studies were unable to correlate the changes in blood n-3 LCPUFA levels with reduced aggressive behaviour. We collected blood samples in our recent feasibility study and will address this limitation in the proposed multi-centre trial. Second, the measure of aggression used within the two previous studies, in-prison reprimands, is influenced by variability both within and between institutions with respect to rules and tolerance (Byrne MK, Cortie CH, Meyer BJ. The Inmate Behaviour Observation Scale (IBOS): A measure of prisoner aggression and violence based on inmate case notes, in preparation/forthcoming). In this study, we used the IBOS, which more specifically targets aggressive behaviour and is less susceptible to institutional variation (Byrne MK, Cortie CH, Meyer BJ. The Inmate Behaviour Observation Scale (IBOS): A measure of prisoner aggression and violence based on inmate case notes, in preparation/forthcoming).

We conducted a feasibility/pilot study at the South Coast Correctional Centre (SCCC) in Nowra, NSW, in 2013 in adult male prisoners [38]. The sample was restricted to male prisoners for both pragmatic and scientific reasons. With regard to the former, the number of female inmates is greatly fewer than male inmates. As such, the task of recruiting a sufficient sample of female offenders for both the purposes of within-group and between-group comparisons was deemed to be difficult to achieve. More importantly perhaps, the literature suggests that female prisoners tend to internalise their anger [39] and that the primary antecedents of female offending are predominantly environmental challenges [40]. In addition to this, there is evidence that the metabolism and bioavailability of omega-3 fatty acids differ between males and females, a variable that would influence the interpretation of results [41–43].

Baseline data showed negative correlations between blood n-3 LCPUFA levels and measures of aggressive behaviour [7]. We have shown that conducting a supplementation trial in a Correctional Centre is feasible [38] and the study design for the multi-centre trial is based on these recommendations [38]: (1) to collaborate with, and receive support from the Department of Correctional Services; (2) the need to provide incentives to prisoners to aid in recruitment; (3) having an external pathology provider take the blood samples; (4) a designated Correctional Officer as a Project Officer to ensure compliance; (5) the need to restrict buy-ups of cans of tuna and n-3 LCPUFA supplements to reduce potential confoundings; (6) the Correctional Officers require training on how to score the IBOS and how to complete the questionnaires; and (7) the inclusion and exclusion criteria need to ensure that participants are likely to remain in the study for the study duration, are aggressive at baseline (IBOS of 1 or greater) and have an omega-3 index below 6% [38].

## Objectives {7}

### Aims

#### Primary aim

To determine if n-3 LCPUFA supplementation reduces aggressive behaviour in adult male offenders in the Correctional Centres using a randomised, double-blind, placebo-controlled intervention and translate these outcomes into policy and practice.

#### Secondary aims

To estimate the impacts of n-3 LCPUFA supplementation on attention deficit disorder behaviour, depression and anxiety, impulsiveness, engagement in and outcomes of rehabilitation programmes, recidivism and physical health (quality of life and muscle strength).

### Hypothesis

#### Primary hypothesis

We hypothesise that 16 weeks of n-3 LCPUFA supplementation will result in significant reductions in aggressive behaviour compared to placebo alone.

#### Secondary hypotheses

We also hypothesise that 16 weeks of n-3 LCPUFA supplementation will result in significant reductions in institutional reprimands and improvements in attention deficit disorder behaviour, depression and anxiety, impulsiveness, engagement in and outcomes of rehabilitation programmes, recidivism and physical health (quality of life and muscle strength).

The null hypothesis is that there will be no difference between the treatments and controls.

## Trial design {8}

The study design is a multi-centre, double-blind, superiority, randomised controlled trial with a 1:1 allocation between experimental and placebo groups.

## Methods: participants, interventions and outcomes

### Study setting

The study will take place in New South Wales and South Australia in Australia in at least 6 Correctional Centres. The Correctional Centres contain between 400 and 1000 prisoners including minimum, medium and maximum security. List of study sites can be obtained in the trial registration (http://www.anzctr.org.au/trialSearch.aspx) and enter ACTRN12618001665224.

#### Public title

The public title of this trial is ‘Does omega-3 supplementation reduce aggressive behaviour in adult male prisoners?’

#### Summary results

Summary results will be listed on the ANZCTR databases once published.

#### Individual clinical trial participant-level data (IPD) sharing statement

The study authors plan to share de-identified IPD underlying published results. Data will be available after all researchers involved in this trial have finished analysing and publishing the data. The de-identified data will be available following the last publication from our research group. No end date is yet determined. Data will be available on request from the study’s CIA BJM (lead and corresponding author) on a case-by-case basis.

All items from the WHO Trial Registration Data Set are available on the ANZCTR databases and within this manuscript.

Based on our successful model from the feasibility study [38], one Correctional Centre Officer per site will be seconded to the research project and they will take on the role of trial coordinator, referred to as Project Officers. Project Officers will be trained at the University of Wollongong prior to the commencement of the trial. Project Officers will determine the 8-week baseline IBOS to identify those prisoners with IBOS scores of greater than 0 and invite them to participate in the intervention trial.

### Eligibility criteria {10}

The participants are adult (aged 18 years or older), male prisoners residing in Correctional Centres.

**Fig. 1:**
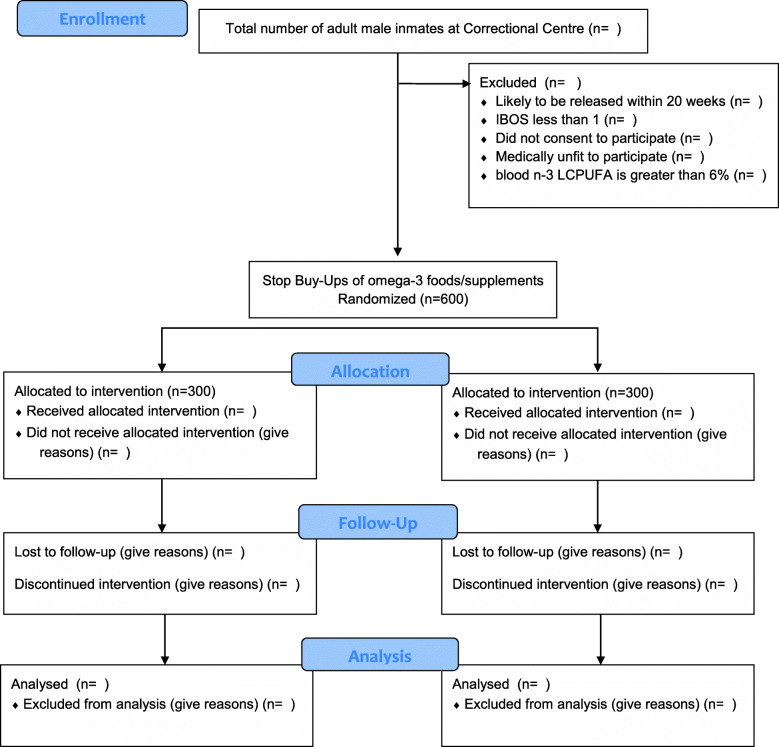
CONSORT 2010 flow diagram

Figure 1 shows the flow diagram for screening, eligibility and randomisation. Inclusion criteria are male prisoners with a baseline IBOS score of greater than 0 and erythrocyte omega-3 index less than 6% of total erythrocyte fatty acids. The IBOS score is explained below.

Exclusion criteria include (1) those prisoners due to leave prison within 16 weeks, (2) prisoners with a baseline IBOS equal to or less than 0, (3) prisoners on blood thinning agents and/or not medically fit and (4) prisoners with an erythrocyte omega-3 index of greater than 6% of total erythrocyte fatty acids.

### Who will take informed consent? {26a}

A potential participant is approached for enrolment in the study if they meet (i) the release date (i.e. will be in the Correctional Centre for the duration of the study) and (ii) the baseline IBOS score (i.e. a score of 1 or greater, indicating aggressive behaviour). The Project Officer will approach these prisoners for enrolment into the study and will take written informed consent. Consent will be sought using scripts incorporating teach-back techniques to ensure participants understand the process. These scripts were developed in consultation with Correctional Officers and a communications specialist to ensure clarity and accuracy. For example, the script regarding consent for the Project Officers to access medical records is ‘The first step is that we need to check your medical records, as there might be a risk involved if you are on medications that thins your blood. You might have to be excluded from the study at this point. We have to ensure all participants are medically approved to take part’ with the teach-back question of ‘Can you tell me why we need to check your medical record?’ Participants will be given up to a week to consider consenting to the study.

### Additional consent provisions for collection and use of participant data and biological specimens {26b}

Additional consent will be sought from the study participants in terms of the storage of their blood samples (bio-banking) and collection of blood samples for future genetic analysis. The study participants will receive separate participant information sheets and consent forms, and the Project Officer will obtain written informed consent using scripts and the teach-back method. Future research may be conducted using these stored blood samples after obtaining the necessary ethics approval.

## Interventions

### Explanation for the choice of comparators {6b}

The algal DHA-O capsules are ~ 1 g with 509.2 mg/capsule DHA/EPA (i.e. DHA 324 mg/capsule and EPA 185.3 mg/capsule), hence providing a daily dose of 1091 mg omega-3 (694 mg DHA and 397 mg EPA). No intervention has assessed blood levels of omega-3; hence, the use of a placebo is permitted [44]. The placebo capsules are a corn/soy oil blend and are identical in size and colour to the active capsules.

### Intervention description {11a}

The Correctional Officer on secondment as the Project Officer will deliver the supplements to the enrolled prisoners on Mondays, Wednesdays and Fridays, and they will watch participants swallow the supplements to ensure compliance. Supplements will be provided over a 16-week duration, the average supplement duration of the 2 previous studies [2, 3]. Baseline and post-intervention measures include IBOS, institutional records of reprimands or misconduct, questionnaires and measure of muscle strength, and blood samples to measure the erythrocyte omega-3 index levels will also be used as a measure of compliance. The questionnaires are demographic (in house), Aggression Questionnaire (AQ) [45]; Browns Attention Deficit Disorder Scales (BADDS) [46]; Conners’ Attention Deficit Hyperactivity Disorder (ADHD) [47]; The Barratts Impulsiveness Scale-11 (BIS-11) [48]; Depression, Anxiety, Stress Scale (DASS-21) [49]; Wellness Questionnaire - the Short Form-36 [50, 51]; plus customised questions on sleep.

The prisoners will also be asked at the end of the trial which treatment they thought they were on.

### Criteria for discontinuing or modifying allocated interventions {11b}

Any report of an adverse event provided by the on-site Project Officer will be evaluated by DG (clinical academic forensic psychiatrist with expertise in clinical research drug trials), and if significant, the participant will discontinue participation. Participants may also withdraw from the study at any time, and if desired consulted prisoner medical services of any medical needs.

### Strategies to improve adherence to interventions {11c}

The Correctional Officer on secondment as the Project Officer will deliver the supplements to the enrolled prisoners and watch them taking the supplements to ensure compliance and therefore monitoring adherence to the study protocol. Adherence will be recorded for each week, with reasons for a lack of adherence recorded. Changes in participant’s omega-3 levels will also be used to determine adherence to treatment.

### Relevant concomitant care permitted or prohibited during the trial {11d}

There are no additional requirements or changes to the participants’ routine other than consumption of the capsules and restriction to buy-ups, as described above. Participants will continue to attend all their scheduled activities such as work and education during the intervention.

Due to the ongoing SARS-Cov-2 pandemic, we, as well as, each Correctional Centre site have developed their own COVID19 safe plan. We submitted these COVID safe plans through an amendment to our ethics committees, and we have received approval from Our University of Wollongong Work Place and Health Safety & Ethics Committees.

### Provisions for post-trial care {30}

There are no expected harms resulting from the trial. All participants will be provided with a post-trial educational package to enable informed independent decisions on the maintenance of dietary/supplemental use of n-3 LCPUFA.

## Outcomes {12}

### Primary outcome measure

Aggressive behaviour will be assessed by the IBOS (Table 1) reported as a single measure, in addition to the number and severity of reprimands with both measures explained below.

**Table 1:** Summary of the study outcome measures and methods

Variables	Methods	Who completes the task?
Primary outcome measure		
Aggressive behaviour	Converting routinely collected case notes into a numerical score of aggressive behaviour using the IBOS (Byrne MK, Cortie CH, Meyer BJ. The Inmate Behaviour Observation Scale (IBOS): A measure of prisoner aggression and violence based on inmate case notes, in preparation/forthcoming)	Correctional Officer on secondment as a Project Officer
Secondary outcome measures		
1. Aggressive behaviour	Counting the routinely collected reprimands into a numerical score and noting them as aggressive or non-aggressive	Correctional Officer on secondment as a Project Officer
2. Questionnaires		
Aggressive behaviour	Aggression Questionnaire [45]	Study participant with or without assistance from the Project Officer
Attention deficit disorder	Brown Attention Deficit Disorder Scales (BADDS, [46])	Study participant with or without assistance from the Project Officer
Attention deficit hyperactivity disorder	Conners’ Attention Deficit Hyperactivity Disorder (AHDH, [47])	Study participant with or without assistance from the Project Officer
Depression and anxiety	21-item Depression and Anxiety Stress Scale (DASS, [49])	Study participant with or without assistance from the Project Officer
Impulsivity	Barratt Impulsiveness Scale (BIS-Brief, [48])	Study participant with or without assistance from the Project Officer
Demographic questionnaire	In-house demographic questionnaire	Study participant with or without assistance from the Project Officer
Health questionnaire	SF-36 [50, 51] general health questionnaire, with additional questions on sleep	Study participant with or without assistance from the Project Officer
Secondary outcome measures		
3. Muscle strength	Dynamometer [52]	Correctional Officer on secondment as a Project Officer
Tertiary outcome measures		
1. Improved engagement in rehabilitation programmes	Completion of programmes and attrition of programmes	Manager of Offender Services & Programs (MOSP)/Manager Offender Development (MOD) will assist the Project Officer
2. Recidivism		CIG DG and Corrective Services Head Office
Other assessments		
Compliance	Erythrocyte fatty acid analysis [7]	CIA BJM and Research Fellow CHC
	Compliance as noted by the Project Officer	Correctional Officer on secondment as a Project Officer

#### Inmate Behaviour Observation Scale (IBOS)

Actual instances of hostile and aggressive behaviour are routinely recorded as prisoner incident reports, where an officer considers the behaviour substantial enough to warrant sanction. However, the intensity of such behaviour required to warrant sanction fails to capture the range and diversity of hostile and aggressive behaviours and thus results in a low incidence rate. Further, different jurisdictions have developed different metrics for the classification of behavioural disorder. Thus, (Byrne MK, Cortie CH, Meyer BJ. The Inmate Behaviour Observation Scale (IBOS): A measure of prisoner aggression and violence based on inmate case notes, in preparation/forthcoming) developed a generic behavioural observation rating scale (IBOS) for use across jurisdictions. The IBOS is a 7-point scale which classifies prisoner behaviours across an 8-week period using data derived from individual prisoner case notes. Case notes are routinely recorded by custodial and non-custodial staff in the Offender Integrated Management System (OIMS) database to report and capture a majority of significant observations and interactions with prisoners. A score of − 1 is applied to all instances recorded in that week of *pro-social behaviour*, while a score of 0 is given if there were no behaviours of significance recorded. Thereafter, instances of hostile/aggressive behaviour are scored from 1 (non-compliant) to 5 (physically aggressive), with each level of hostility/aggression operationally defined and illustrated by examples. Thus, higher scores are associated with greater levels of aggressive behaviour. Inter-rater reliability of the IBOS was 0.844 for repeat raters (Byrne MK, Cortie CH, Meyer BJ. The Inmate Behaviour Observation Scale (IBOS): A measure of prisoner aggression and violence based on inmate case notes, in preparation/forthcoming). The IBOS is a validated tool that can be used across jurisdictions. The IBOS is significantly correlated with institutional records of misconduct or reprimands (Byrne MK, Cortie CH, Meyer BJ. The Inmate Behaviour Observation Scale (IBOS): A measure of prisoner aggression and violence based on inmate case notes, in preparation/forthcoming) which are the most common measure of aggression in prison studies. The IBOS has been found to significantly correlate with both the Aggression Questionnaire (AQ) and the Brown’s Attention Deficit Disorder Scales (BADDS) [7], and given that the AQ and the BADDS are also significantly correlated [53], this adds to its construct validity.

#### Number and severity of reprimands

If an act of aggressive behaviour warrants a reprimand, they are recorded in the OIMS database by Correctional Officers. The reprimands will be extracted from the OIMS system by the Project Officer and classified as violent or non-violent cases.

### Secondary outcome measures

#### Psychological assessments—questionnaires (Table 1)

With respect to antisocial behaviour and omega-3 supplementation, the measurement of psychological change will focus on both the behavioural and the psychometric expression of aggression. Previous research [2] has used the *Aggression Questionnaire* (AQ) [45] to assess cognitive changes in aggression. The AQ is a self-report inventory that screens adults for aggressive tendencies, providing sufficient detail to enable treatment outcome measurement.

The benefits from essential fatty acid supplementation with ADHD are most likely with the inattentive subtype [54]. Recent models of attention deficit disorders (ADDs) have suggested that the core symptoms of ADDs are essentially cognitive [55]. Emotions have been identified as being one of the cognitive functions affected by ADDs, with the management of frustration and anger often impaired [56]. Such deficits are assessed by the *Brown Attention Deficit Disorder Scales* [46]. The BADDS has been used for ADD screening as well as the monitoring of treatment effectiveness of ADD and has been reported to have good construct and internal validity as well as discriminative power [57]. *Conners’* ADHD scales [47] have been well-validated and widely used in ADHD research including a range of omega-3 studies [33]. The 21-item Depression and Anxiety Stress Scales (*DASS-21* [49];) will be used to assess depression, anxiety and stress. Barratt Impulsiveness Scale (*BIS-Brief*) will be used to assess impulsiveness [48]. The Project Officers will assist in the delivery of psychological tests (*AQ*, *BADDS*, *Conner’s ADHD*, *DASS-21* and *BIS-Brief*) at baseline and post-intervention.

#### Changes in engagement in rehabilitation programmes and changes in scores of outcome measures (Table 1)

The Manager of Offender Services & Programs (MOSP)/Manager Offender Development (MOD) in each centre will play an integral part in the rollout and overall coordination. The MOSP/MOD has responsibility for case management in each centre and the selection of prisoner suitability for programmes. The Project Officers will liaise with the MOSP/MOD and collect the data on improved engagement in programmes. Data are routinely kept on programme completion and attrition, which will be used as a proxy for engagement.

#### Post-release benefits: changes in recidivism (Table 2)

The NSW Bureau of Crime Statistics and Research’s Reoffending Database (ROD) was developed to investigate reoffending. It is an identified, internally linked dataset containing records of all finalised court appearances in the Local, District and Supreme Courts of NSW since 1994. An individual’s recorded aliases are also included. The internal matching process of this database has been previously validated. Key variables include sex, month and year of birth and offending history (e.g. principal offence type, offence date, median sentence ranking [offence seriousness], number of concurrent offences, number of court appearances with at least one prison penalty, number of court appearances for a violent offence). The ROD will be used to measure categories of post-release of reoffending, given this database is a sensitive measure which is independent of public policy and incarceration rates. The number, severity and frequency of re-offences will be quantified. Recidivism rates for categories of offence will be calculated over a 2-year period for those participants who have been released. Potential for long-term benefits of omega-3 supplementation has been previously suggested in a double-blind randomised controlled trial where with a 12-week omega-3 intervention, followed by no supplementation for 12 months, 5% in the omega-3 group while 28% in the control group transitioned to psychotic disorders [58].

**Table 2:**
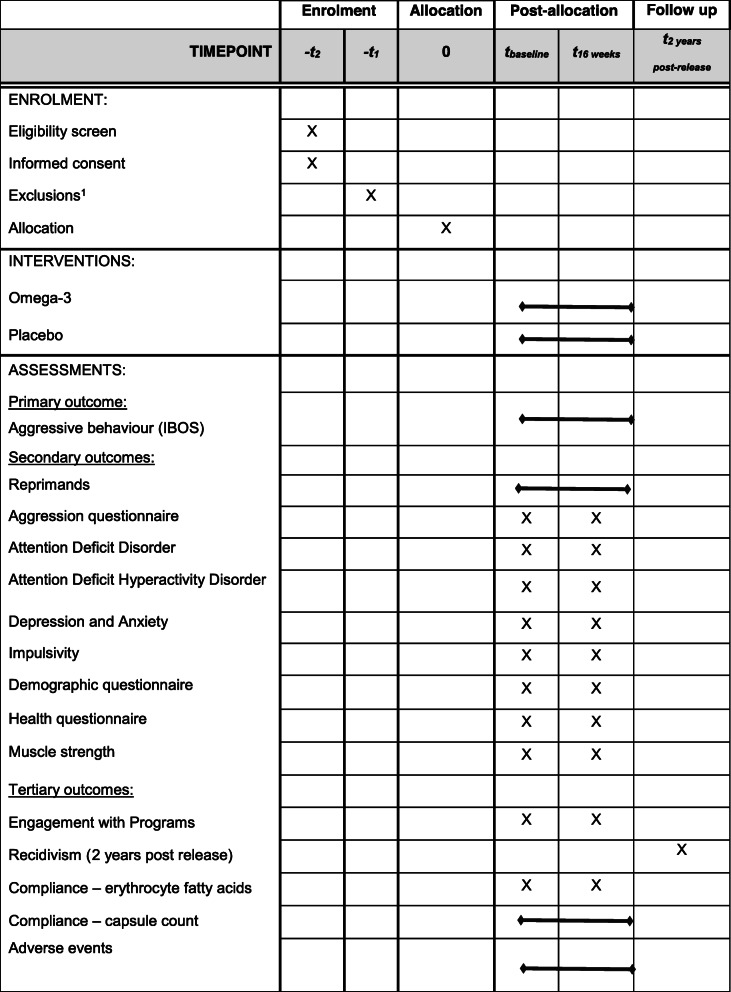
Schedule of enrolment, intervention and assessments

^1^IBOS less than 1, blood clotting disorders, blood thinning medication, known medical condition, omega-3 index greater than 6% of total erythrocyte fatty acids

#### Health and quality of life (Table 1)

The validated SF-36 Quality of Life Questionnaire (adapted to suit prisoners) will be used to assess health-related quality of life. The adapted versions will include 3 questions on sleep. Muscle strength will be assessed by hand grip strength [59].

#### Assessment of blood samples (Table 1)

All blood samples will be transported from each site to the University of Wollongong (UOW). The erythrocytes will be stored in a minus 80 °C freezer at UOW prior to analysis. The erythrocyte fatty acids will be analysed in CIA BJM’s laboratory using standard techniques [7].

### Translation into policy and practice

Adapting ‘what works to promote evidence based practice’ [60], the overall objective is to create a scenario where, nationally, ex-prisoners continue adherence to omega-3 supplementation post-release, with the expectation that in doing so, maintenance of reduced recidivism rates will be achieved. For this to occur, there needs to be:

1. A framework for national incorporation of dietary supplementation within prison systems
2. An educational programme for prisoners on the benefits of omega-3 supplementation
3. Adherence to treatment practices and policies to overcome obstacles to continued effective ingestion of omega-3, including issues associated with cultural sensitivities.

#### A framework for national dietary supplementation

If a robust evidence base is established for the efficacy of omega-3 supplementation on behavioural improvement, attention enhancement and engagement in correctional rehabilitation, the first translational challenge is to disseminate the programme nationally. This will require, in the first instance, a framework for the dissemination of supplements across jurisdictions, accounting for health considerations (such as prisoner use of blood thinning agents as part of their medical treatment), leveraging the Corrective Services Advisory Committee and, in particular, the network of AI LG. To do this, dietary outcomes would need to first be linked to extant policies on prisoner nutrition (such as the Corrective Services Industries Food Services’ Nutrition Policy Statement, 2015), indicating the obligation for a minimum intake of omega-3-rich foodstuffs. Next, stakeholder dialogue of a National Round Table (NRT) of Corrective Services policy makers through the Corrective Services Administrators Council to agree on a Policy Statement on the minimum supplementation schedule to achieve clinical outcomes commensurate with the evidence base. A briefing document, containing all relevant information, would be provided to the stakeholders and real-time feedback sought to ensure a high response rate. Following the NRT, the research team could collaborate with key stakeholders to develop a tool-kit for rollout in each jurisdiction for ‘system readiness’ by involving and mobilising ground-level stakeholders, human resources and service infrastructure requirements, management systems and monitoring and evaluation systems.

#### Educational programme for prisoners

To effectively implement the programme, prisoners will need to be both consulted and educated about the range of health benefits associated with omega-3 supplementation. This will involve consultation with prisoner representative groups and cultural sensitivity advisors to establish a protocol for the dissemination of information. The delivery of the educational package will be modelled on the NSW Corrective Services ‘Healthy Survival Tips’ programme which involves a 4-h education programme on induction to prison with the SAPO (Services and Program Officer); a pre-release programme, integrated into programmes similar to the Corrective Services NSW ‘Nexus’; and the provision of written ‘brochure’ material on ‘Healthy Living Outside Gaol’ where omega-3 supplementation may be integrated into general health advice. Given the low level of formal education received (results from feasibility trial) and the associated lack of health literacy skills, in addition to the aforementioned educational packages, prisoners will also educate themselves and champion findings. For example, in the feasibility study, the prisoners did a rap video ‘Omega-3s are the key, they are good for you and good for me’.

#### Adherence to treatment practices and policies

Adherence to health interventions is a perennial problem across health contexts and is likely to be an issue with omega-3 supplementation. A broad range of adherence interventions exist. First and foremost, the fiscal cost of maintenance post-release will need to be addressed. To manage this, Commonwealth support similar to PBS schemes should be established. This would require liaison with the Federal Minister and/or his/her representatives informed by evidence of joint health and social system effect of omega-3 supplementation on violence and recidivism rates and related economic consideration of expected downstream health and social systems savings relative to investment [61–64] in omega-3 supplements in prison populations. Advocacy for a ‘Health Care Card’ system where purchase of prescribed omega-3 supplements by former prisoners is subsidised could be sought. Additionally, with positive findings post-release, supervision policies could be negotiated at the federal and state level (incorporated in discussions under ‘National Framework’ above) whereby adherence issues are resolved at a prisoner by prisoner level. A brief training video would also be disseminated to Probation and Parole offices via YouTube to enhance adherence management skills relevant to omega-3 intake.

#### Authorship

It is anticipated that all authors on the protocol paper will also be authors on the primary outcome paper.

### Participant timeline {13}

Participant timeline is shown in Table 2.

### Sample size {14}

Our study is a randomised controlled trial powered to show a difference between treatment and placebo control of 25% in the proportion of participants experiencing a reduction in aggressive behaviour as measured by a change in the IBOS. Given the nature of the prison populations, it is likely that there will be an effect of prison in the analysis, and therefore, a design effect accounting for the intra-cluster correlation is incorporated in the sample size estimation with a conservatively estimated ICC of 0.03. Based on our pilot data, we anticipate that attrition will likely be high (~ 40%) with the sample size for recruitment appropriately inflated to account for this. Overall, a total sample size of 600 with 360 completing the study enables a difference of 25% to be detected with 80% power and an alpha level of 0.05.

### Recruitment {15}

We were able to recruit 136 prisoners in only 1 day at the SCCCfor our feasibility study. The success of this recruitment was primarily due to what the trial was able to offer: a perception of increased muscle strength [52] and receiving compensation for inconvenience ($30 at baseline and $50 post-trial). Taking into account the inclusion criteria of this multi-centre trial, of those 136 prisoners, 34 would be excluded due to high omega-3 index (data from our feasibility study). Given that recruitment will occur at multiple centres with one or two cohorts per site as necessary, we anticipate comfortably meeting our targets of 600 prisoners and 360 completions.

## Assignment of interventions: allocation

### Sequence generation {16a}

Participants will be block randomised (according to their IBOS, age and baseline omega-3 index) within each treatment centre to one of the 2 study arms. The randomisation will be performed by Chief Investigator MB (biostatistician) using the RALLOC command in STATA V12 or higher (College Station, TX).

### Concealment mechanism {16b}

Allocation will occur following the assessment of participant eligibility. Participants will be allocated to group A or group B by block randomisation. A person independent of the study will allocate the control and experimental cohorts to group A or B and will hold the allocation key in a sealed envelope. Supplement jars will not be labelled with group identification, only participant’s identification such that each study participant has their own allocated supplements without the labels ‘A’ or ‘B’ on them. Participants and the Project Officer will be blinded to the intervention group allocation throughout the study.

### Implementation {16c}

The generation of allocation sequence will be performed by Chief Investigator MB (biostatistician) using the RALLOC command in STATA V12 or higher (College Station, TX). One designated Project Officer on secondment to the research trial at each of the sites will enrol the potential participants at their respective sites.

## Assignment of interventions: blinding

### Who will be blinded {17a}

Project Officers administering the supplements, participants receiving the supplements and the people assessing the outcomes and analysing the data will be blinded to interventions. The randomisation list will be managed through an independent third party who will keep treatment allocation totally blinded.

### Procedure for unblinding if needed {17b}

It is anticipated that unblinding the trial will not be necessary. Revealing a participants’ allocation intervention during the trial will not be necessary.

## Data collection and management

### Plans for assessment and collection of outcomes {18a}

Correctional Officers on secondment to the research project as Project Officers will attend a 3-day training workshop at the University of Wollongong. They will be educated on research rationale and the anticipated outcomes. They will be trained in (1) how to translate the written case notes into a numerical IBOS score; (2) how to approach potential study volunteers in a culturally sensitive way; (3) the process for baseline and post-intervention assessments (including the procedures for blood collection, and how to conduct the questionnaires and use the dynamometer to measure muscle strength); (4) how to deliver the capsules and check for compliance; and (5) how to enter the data in the database.

A manual outlining the standard procedures for screening, recruitment, data collection and completing the study will be given to Project Officers.

The IBOS is a validated tool that can be used across jurisdictions and has high inter-rater reliability. The questionnaires are all validated and are used frequently in this area of research. The blood omega-3 assessments are routinely conducted in CIA BJM’s laboratory at the University of Wollongong.

### Plans to promote participant retention and complete follow-up {18b}

Project Officers will routinely discuss participant experience of the trial as the capsules are delivered. In doing so, participant indication of a desire to discontinue trial engagement will be discussed and where individual problems associated with participation are identified, the Project Officer will assist the participant to problem solve a solution to enable ongoing participation.

### Data management {19}

The post-doctoral person at UOW, CHC, will be responsible for data collation and ensuring data quality control checks under the supervision of CIA BJM and CIB MKB.

The on-site data will be collected using excel templates: one identifiable and one de-identifiable. The identifiable dataset will be stored on a secure server at Correctional Centre Head Office and will ultimately be used to assess recidivism. The study participants will be given a unique identifier that incorporates their Correctional Centre and a three-digit number and all intervention data will be captured in this de-identified dataset. This de-identified data will ultimately be imported into RedCap (Research Electronic Data Capture [65, 66]) and hosted at the University of Wollongong local servers and external hard drive. The de-identified dataset will be protected by a password or RedCap’s data access groups. Access to the dataset will be on a needs basis and certainly provided to our biostatistician who will be primarily responsible for the data analysis. Data quality control checks will be carried out during recruitment and post-interventions, and prior to analysis. Data quality checks will include range checks, duplicate checks, missing data checks and checks for data entry errors.

### Confidentiality {27}

The Correctional Officers seconded to the research study as Project Officers will enter data into two datasets: (1) a re-identifiable dataset that contains personal information and the study participants’ unique identifier code and (2) a de-identified dataset, where all the collected data from the research trial is entered. The re-identifiable dataset will be held in Correctional Services Head Office and will be used for assessing rates of recidivism. The de-identified dataset will be held with the researchers. The data files will be kept in secure locations at the UOW with appropriate back-up.

### Plans for collection, laboratory evaluation and storage of biological specimens for genetic or molecular analysis in this trial/future use {33}

Upon written consent, the potential study participant’s medical records will be checked to determine if they are taking blood thinning medications or are physically unfit to take part in the research trial. Chief Investigator DG who has medical qualifications will assess the potential study participant’s medical eligibility. A baseline blood sample will be taken from the eligible study participants by qualified staff from Pathology centres. The blood samples will be analysed for omega-3 index in Chief Investigator BJM’s laboratory at the University of Wollongong. Those prisoners with an omega-3 index of less than 6% will be eligible to commence the intervention trial, on the provision that they forgo purchase of additional fish (tuna, salmon) and n-3 LCPUFA supplements from their casual ‘buy up’ for the duration of the trial. They will be offered compensation for inconvenience: $30 after the baseline assessments and $50 after the completion of all the post-intervention assessments.

Blood samples will be stored in a BioBank from those prisoners that consent to their blood samples being stored. Future research may be conducted using these stored blood samples after obtaining the necessary ethics approval.

## Statistical methods

### Statistical methods for primary and secondary outcomes {20a}

In the simplest case, this study is designed to determine the difference between groups as proportion of responders to the treatment allocation. This analysis will be performed using a generalised linear mixed model adjusted for clustering by prison. IBOS scores will also be analysed using a form of generalised linear mixed model or generalised additive mixed models depending on the final distribution of the IBOS scores. These models also allow for the correlated nature of repeated measures over time and the correlation between prisoners from the same institution. Secondary measures will be analysed using linear mixed, or generalised linear mixed models again depending on the distributional form of the variable. These analyses will be conducted using STATA or R.

The data will also be analysed according to the change in the omega-3 index [24] as recommended by ISSFAL [24].

Our policy implementation is based on the primary outcome measure, the IBOS. The secondary outcome measures are hypothesis generating.

### Interim analyses {21b}

Not applicable, as there is no need for interim analyses.

### Methods for additional analyses (e.g. subgroup analyses) {20b}

A priori, a subgroup per protocol analysis is planned for those participants deemed to be compliant with treatment. Subgroup analysis that will be considered include 80% compliance to capsule regime, age as a covariate, per change in omega-3 as recommended by the ISSFAL [24] and the Aggression Questionnaire—high vs low dispositional aggression.

The covariates that might be adjusted for include baseline DASS scores and SF-36 scores. It is acknowledged that reactive aggression is influenced by depression, anxiety and stress and this will be identified through the DASS. The dispositional ‘satisfaction’ will be assessed via the SF-36. With respect to ADHD, we hypothesise a mediating role of ADHD in aggressive behaviour.

### Methods in analysis to handle protocol non-adherence and any statistical methods to handle missing data {20c}

The primary analysis with be on an intention-to-treat basis. A per-protocol analysis will be performed on the subgroup deemed to be compliant with the trial protocol. Longitudinal analyses will be performed using restricted maximum likelihood, and therefore, all participants providing partial data will be included in the analysis.

### Plans to give access to the full protocol, participant-level data and statistical code {31c}

Data and blood samples will be retained for the potential to analyse additional health benefits including anti-inflammatory effects, nutritional, metabolic and oxidative markers on the cohort subject to funding. The data and blood samples will be retained for the potential use of longer term outcomes on the cohort. The State Records requirement for research data states that samples should be stored for a minimum of 20 years after the project is completed. Samples will be stored in the NSW Health Statewide Biobank in Sydney. There are plans for sharing the de-identified data, after the original researchers have completed all the data analysis and published the findings.

The NHMRC encourages data sharing and it is envisaged that the data sharing will commence after our study is completed—hence, the necessary approval from study participants for this to occur up front as to seek approval from them in the future would be impossible. The data that will be shared will be completely de-identified data.

Future use of data could include longer term recidivism outcomes. Should the researchers wish to utilise the facility of the re-identifiable dataset, they would need to consult with the custodians of that dataset, i.e. Corrections in each state.

Follow-up research could include the influence of this research on the development of policy and practice and the rollout and adherence to policy.

Data will be available after all researchers involved in this trial have finished analysing and publishing the data. Data may become available to only those researchers who provide a methodologically sound proposal, and this will be determined on a case-by-case basis at the discretion of the Principal Investigator.

Secondary analysis of data could include the link between ADHD and criminal infractions.

## Oversight and monitoring

### Composition of the coordinating centre and trial steering committee {5d}

The University of Wollongong is the coordinating centre. The Principal Investigator and the second Principal Investigator are largely responsible of the running of the research project and supervision of the post-doctoral fellow.

A steering committee comprising of all Chief Investigators and the Governors (or their nominees) from Correctional Centres will meet on a regular basis to discuss the progress of the trial for each site. A regular quarterly newsletter will be circulated to all Chief Investigators, Associate Investigators, Governors and partner organisations informing regarding the progress of the research project.

### Composition of the data monitoring committee, its role and reporting structure {21a}

Data monitoring will be undertaken by the trial management group: CIA, CIB and the post-doctoral research fellow. Given that the researchers are blind to allocation, data monitoring will occur solely to ensure data quality and adherence to the study protocol. Data monitoring in terms of recruitment rates will be undertaken by the funding bodies: the National Health and Medical Research Council and DSM Nutritional Products.

### Adverse event reporting and harms {22}

Adverse events will be collected by spontaneous report by the Project Officer. All adverse events will be reported in trial publications. Any adverse medical outcomes (it is anticipated that these, if any, will be minor, e.g. bruising after blood collection) will be recorded by the Project Officer using the Adverse Events form. The Project Officer will notify the CIA BJM of these adverse events as they occur who will discuss the adverse outcomes with CIG DG and they will react as necessary and responsibly. All adverse events will be reported to DSM when they occur and definitely at the end of the study. Usual corrective services protocols for prisoner health will continue.

### Frequency and plans for auditing trial conduct {23}

Regular meetings with Governors of Correctional institutions (or their nominees) will occur to ensure that the trial is conducted as detailed in the protocol.

### Plans for communicating important protocol amendments to relevant parties (e.g. trial participants, ethical committees) {25}

If there is a need to amend the study protocol, the relevant parties will be notified accordingly. Ethics approval will be obtained if and when necessary.

## Dissemination plans {31a}

The University of Wollongong will provide a Final Study Report to DSM 6 weeks before the close of study for their approval prior to the publication of results. Outcomes will be published in peer-reviewed international scientific journals and presented at national and international conferences.

The University of Wollongong research report on the intervention will be made available to all participating and non-participating Correctional Centres in Australia and their administrative bodies.

For translation into policy and practice:

For dissemination of positive findings to achieve both reduced institutional violence and potentiate reduced recidivism, there needs to be:

1. A framework for national incorporation of dietary supplementation within prison systems
2. An educational programme for prisoners on the benefits of omega-3 supplementation
3. Adherence to treatment practices and policies to overcome obstacles to continued adequate ingestion of omega-3, including issues associated with cultural sensitivities

## Discussion

At least 45% of prisoners in Australia are incarcerated for violent offences [67]. Approximately 46% of prisoners have experienced a mental health disorder [68]. The rate of prisoner on prisoner assault reported in NSW and QLD prisons is 34% [69].

Prisoners cost the nation $2.6 billion in 2013–2014 after expenses, with the average daily cost per prisoner exceeding the average Australian’s daily income [70]. Should the intervention reduce aggressive behaviour by 25%, the estimated impact upon institutional violence would be an 8.5% reduction based on extant assault prevalence rates (34%). Some estimations of cost savings across prison populations nationally would suggest up to $236 million/year (at a conservative $8500 per aggressive prison patient prevented). This intervention also has potential to enhance overall prisoner health outcomes and if omega-3 use is maintained post-incarceration to reduce violent recidivism.

Our feasibility study showed that prisoners are 4.3 times more likely to have an IBOS of > 2, if they are below the cutoff of 6% omega-3 index [38] but this does not show causality. This multi-centre trial is adequately powered to demonstrate the effect of n-3 LCPUFA on aggressive behaviour in adult male prisoners. The strengths of this study include the IBOS, measuring n-3 LCPUFA levels in the blood and, after demonstrating efficacy, rolling out into policy and practice and the health economics. The IBOS eliminates cross- and intra-jurisdictional variations in reprimands, robustly accounting for prisoner aggressive behaviours. The blood n-3 LCPUFA assessments will verify that the reduction in aggressive behaviour is related to an increase in n-3 LCPUFA. Once we demonstrate efficacy, we will translate into policy and practice across all jurisdictions in Australia. Given the high costs associated with prisoner aggressive behaviour, substantial cost savings are expected.

Our research team has the track record to navigate studies through the ethics approval process, as evidenced by the feasibility studies and publication outcomes [7, 53]. This research will be carried out in accordance with the Declaration of Helsinki [44] and the Australian National Statement on Ethical Conduct in Research Involving Humans [71]. This research will follow the CONSORT guidelines and is registered with the Australian New Zealand Clinical Trials Registry.

We are aware of ethical considerations in research with Aboriginal and Torres Strait Islander people, including reciprocity, respect, equality, responsibility, survival and protection, spirit and integrity. Therefore, we believe that in the spirit of these considerations, it is important for Indigenous people in the institution to be given the same opportunity to take part in this research. The research does not include a focus on culturally specific issues; all people regardless of ethnicity can potentially derive benefits from the nutrients that are the focus of this research.

Informed consent will be obtained from all study participants and there will be no coercion. Each participant will be provided with a unique study code identifier. The Project Officer at each site will provide each participant with their supplements and supervise them taking the supplements, which will minimise intimidation for access to individual capsules. The Project Officer will keep one data file with the prisoners’ names and their unique study code identifier, hence ensuring confidentiality. All research data will be stored in another de-identified dataset containing the unique study code identifier, hence allaying concerns about blood data being shared.

This trial is a partnership between academic researchers, DSM Nutritional Products, and Correctional Services NSW and the Department for Correctional Services SA. The role of DSM Nutritional Products is to supply the omega-3 (algal oil) and placebo oil capsules as in-kind support, plus provide substantial cash contribution for this research. The role of Correctional Centres in NSW and SA is to provide in-kind support of personnel (MOSP/MOD) who have the responsibility for case management in each centre and the selection of prisoners for suitability for the rehabilitation programmes. The Project Officers (on secondment to this research) will liaise with the MOSP/MOD and collect the data on improved engagement in programmes. The role of the Corrective Services Assistant Commissioners (including AI LG, Deputy Commissioner, Strategic Policy and Planning CSNSW) is to take a lead role from our team to manage the translation into policy and practice as described above.

The expected outcome is that supplementation with n-3 LCPUFA will reduce prisoner aggressive behaviour (as measured by the IBOS and the number and severity of reprimands), reduce aggressive disposition (AQ), attenuate attention deficit disorder behaviours (BADDS), reduce impulsiveness (BIS-brief), improve overall health and wellbeing (SF-36) and enhance rehabilitation programme engagement and gains. Increased and sustained attendance at Correctional Programs should ultimately reduce recidivism. The commitment by our collective team including communication expert and AI LG will ensure translation into policy and practice as described.

The significance of this research is the potential to improve mental health through nutritional supplementation while reducing the incidence of aggressive behaviour. This can contribute to reduced sentencing and time in custody as well as stress and burden on corrective services staff, including costs on corrective services. N-3 LCPUFA are considered to be the building blocks of good mental health, and they provide a low-cost (<$100 per year per person) and low-risk addition to a suite of mental health interventions in correctional centres. The Australian Institute of Criminology estimates that assaults alone cost Australia nearly $2.5 billion a year [72]. Given that between 35 and 41% of prisoners will be a recidivist within 2 years [73], any reduction in ongoing offending behaviour is likely to yield substantial social and economic benefit. The Health economist (CIE SE) will estimate the actual cost saving expected given trial effects observed as part of this project. Furthermore, the findings from this project have the potential for substantially broader benefits across social systems and for society if aggression and violent behaviour and metal health issues of prison populations are reduced.

## Trial status

The protocol version number is OmegaMan2018-002, version 6, 24 August 2018.

Recruitment commenced in April 2019 at one Correctional Centre and the approximate date when recruitment will be completed is December 2021.

## Abbreviations

ADDs: Attention deficit disorders
ADHD: Attention deficit hyperactivity disorder
AQ: Aggression Questionnaire
BADDS: Browns Attention Deficit Disorder Scales
BIS-Brief: Barratt Impulsiveness Scale
CVD: Cardiovascular disease
DASS-21: Depression and Anxiety Stress Scales
DHA: Docosahexaenoic acid
EPA: Eicosapentaenoic acid
IBOS: Inmate Behaviour Observation Scale
ISSFAL: International Society for the Study of Fatty Acids and Lipids
MOSP: Manager of Offender Services & Programs
MOD: Manager Offender Development
NRT: National Round Table
n-3 LCPUFA: Omega-3 long chain polyunsaturated fatty acids
OIMS: Offender Integrated Management System
